# Immunoregulation in human malaria: the challenge of understanding asymptomatic infection

**DOI:** 10.1590/0074-02760150241

**Published:** 2015-12

**Authors:** Vitor R de Mendonça, Manoel Barral-Netto

**Affiliations:** 1 Fundação Oswaldo Cruz, Centro de Pesquisas Gonçalo Moniz, Salvador, BA, Brasil; 2 Universidade Federal da Bahia, Faculdade de Medicina, Salvador, BA, Brasil

**Keywords:** asymptomatic infection, immune response, biomarkers, networks

## Abstract

Asymptomatic *Plasmodium* infection carriers represent a major threat to malaria control worldwide as they are silent natural reservoirs and do not seek medical care. There are no standard criteria for asymptomatic*Plasmodium* infection; therefore, its diagnosis relies on the presence of the parasite during a specific period of symptomless infection. The antiparasitic immune response can result in reduced*Plasmodium* sp. load with control of disease manifestations, which leads to asymptomatic infection. Both the innate and adaptive immune responses seem to play major roles in asymptomatic *Plasmodium*infection; T regulatory cell activity (through the production of interleukin-10 and transforming growth factor-β) and B-cells (with a broad antibody response) both play prominent roles. Furthermore, molecules involved in the haem detoxification pathway (such as haptoglobin and haeme oxygenase-1) and iron metabolism (ferritin and activated c-Jun N-terminal kinase) have emerged in recent years as potential biomarkers and thus are helping to unravel the immune response underlying asymptomatic *Plasmodium* infection. The acquisition of large data sets and the use of robust statistical tools, including network analysis, associated with well-designed malaria studies will likely help elucidate the immune mechanisms responsible for asymptomatic infection.

It is estimated that two-three billion people are at risk of contracting malaria, and nearly one million people die from this disease each year (WHO 2014). The spectrum of malarial disease can range from severe complications to a mild symptomatic infection to an asymptomatic carrier infection. Such distinct manifestations result from a combination of factors, including parasite virulence, host susceptibility, host immune response, disease tolerance mechanisms, and environmental factors (Andrade & Barral-Netto 2011, Medzhitov et al. 2012).

Although there is no standard definition of asymptomatic plasmodial infection (API), individuals with API harbour the parasite as evidenced by positive parasitaemia. However, these individuals do not develop any symptoms during a defined period of time (Andrade & Barral-Netto 2011, Lindblade et al. 2013). API is an significant obstacle to malaria eradication efforts and represents a serious healthcare problem for the following reasons: (i) serve as parasite reservoirs, which allow malarial disease to be maintained within a population over time as they can still transmit*Plasmodium* sp. to uninfected persons (Gouagna et al. 2004, Alves et al. 2005, Schneider et al. 2007, White 2008), (ii) asymptomatic carriers represent a serious risk to blood bank safety as API carriers can transmit malaria through blood transfusions (Najem & Sulzer 2003, Fugikaha et al. 2007, Scuracchio et al. 2011, Anthony et al. 2013, Brouwer et al. 2013), and (iii) human immunodeficiency virus (HIV)-infected individuals with API sometimes exhibit increased viral load, which may enhance HIV transmission and accelerate disease progression and severity in endemic countries (Verhoeff et al. 1999, Whitworth et al. 2000,French et al. 2001, Kublin et al. 2005).

API can be attributed to several factors, including differences among*Plasmodium* sp. and host protective mechanisms. API is frequently associated with older people living in endemic areas as they are likely to have greater exposure to malaria and its vector in endemic settings over time, thus acquiring a partial immunity (Andrade et al. 2009, Ladeia-Andrade et al. 2009, Mendonça et al. 2013). In the same context, individuals who have had several previous episodes of symptomatic malaria are more likely to become asymptomatic carriers upon *Plasmodium* sp. infection (Andrade et al. 2009, Barbosa et al. 2014). Therefore, the immune response underlying asymptomatic infection still needs to be elucidated.

Individuals from endemic regions can acquire partial immunity to malarial parasites, and antidisease immunity may prevent the development of clinical symptoms of disease despite the presence or the number of parasites. Antiparasitic immunity (after a certain age) against *Plasmodium* sp. suppresses parasite load (Day & Marsh 1991, Trape et al. 1994, Daubersies et al. 1996). The immune response in API is often described as disease resistance, which is associated with a reduction in pathogen burden; therefore, this protective mechanism reduces tissue damage and immunopathology related to malarial infection (Medzhitov et al. 2012). In contrast, some individuals can control disease manifestation despite not being able to reduce levels of parasitaemia; this phenomenon is described as disease tolerance (Medzhitov et al. 2012).

Immunity to malaria does not necessarily prevent infection; however, it does limit parasite density and symptoms (Tran et al. 2013). API individuals can remain infected for long periods even though asymptomatic subjects can develop symptomatic disease if they have a dysregulated immune response (Barbosa et al. 2014). Several studies have reported very low parasitaemia in individuals with API (Perkins et al. 2005, Minigo et al. 2009, Andrade et al. 2010b, Villasis et al. 2012), and many of them exhibited subpatent infections (i.e., infections undetected by microscopy) (Barbosa et al. 2014). Asymptomatic carriers who are not diagnosed with conventional malaria are a major challenge for malaria eradication in low-endemicity settings (Bousema et al. 2014). Taken together, these data illustrate the interaction between malarial immunity, parasitaemia, exposure, and malaria outcomes in endemic areas (Fig. 1).

**Fig. 1:**
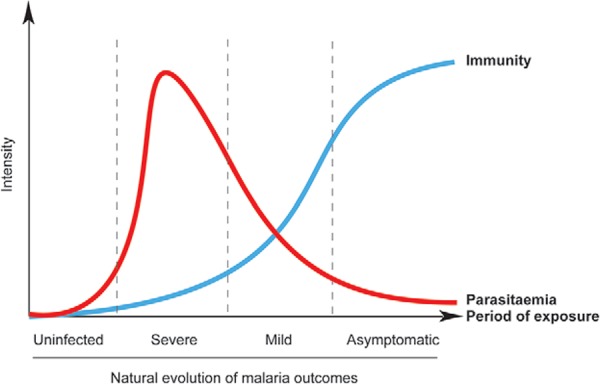
: understanding the natural evolution of malaria outcomes by parasitaemia, immunity, and period of exposure in endemic areas. In endemic settings, the natural evolution of malaria is initiated when uninfected individuals become infected for the first time, usually children who then develop a severe form of the illness. It is known that subjects with severe malaria have high parasitaemias and overall low protective immunity against malaria. In subsequent malarial infections, individuals initiate a more robust immune response against the parasites and exhibit lower levels of parasitaemia and milder forms of this disease. After many years of exposure to malaria and its vector, older people become resistant to malaria by exhibiting higher levels of antiparasitic immunity. Adapted from Andrade and Barral-Netto (2011).

The immune system seems to play a major role in malaria outcomes, and our object herein is to uncover the partial protective immune response to infection in API to unravel the mechanisms of disease resistance. Here, we review both innate and adaptive immune responses to *Plasmodium* infection as well as new approaches to understand API immunity.

Although not the main focus of this review, it is important to highlight that pathogen-related infections can modulate the immune response of individuals with malaria. In this context, asymptomatic infections have been reported to be composed of multiple genetically distinct *Plasmodium* sp. clones; multiclonal infections may be a marker of immunity and confer protection against malaria by inducing a broader immune response and tolerance to infection (Ntoumi et al. 1995, Felger et al. 1999, Smith et al. 1999, Rono et al. 2013). Regarding others pathogens, hepatitis B co-infection has been associated with *Plasmodium vivax*asymptomatic infection and may also boost the protective immune response (Andrade et al. 2011). Additionally, individuals co-infected with *P. vivax* and hepatitis B virus (HBV) have an increased HBV viraemia yet a decreased malaria parasitaemia (Andrade et al. 2011). These patients also have lower levels of pro-inflammatory tumour necrosis factor (TNF) and a lower interferon (IFN)-γ/interleukin (IL)-10 ratio with higher levels of regulatory IL-10 (Andrade et al. 2011). Pre-existent filarial infection also seems to attenuate immune responses associated with severe *Plasmodium falciparum*malaria and protects against anaemia (Dolo et al. 2012). Co-infections with *Ascaris lumbricoides* or*Schistosoma hematobium* exhibit a trend towards a protective effect, whereas infections with hookworm or *Schistosoma mansoni* lead to aggravation of pathology and a higher incidence of malaria (Adegnika & Kremsner 2012, Lemaitre et al. 2014).

Haemoglobinopathies, including haemoglobin S (HbS), haemoglobin C (HbC), and α-thalassaemia, have been associated with protection from malaria (Mendonça et al. 2012a). API children with HbS and persistently positive smears exhibit a reduced median time for conversion to smear-negative responses (spontaneous clearance) than do children without the haemoglobinopathy (Billo et al. 2012). Mechanisms by which haemoglobinopathies may attenuate the pathogenesis of malaria caused by *P. falciparum* include modulation of the inflammatory response and enhancement of cell-mediated and humoural immune responses through pathways that may include haeme oxygenase-1 (HO-1), reduced levels of cerebral chemokines, increased levels of nitric oxide, and higher IgG seroreactivity to *P. falciparum* antigens (Taylor et al. 2013). Other host erythrocyte polymorphisms also seem to influence the susceptibility to malaria. It has been demonstrated that α+-thalassaemia (Oppenheimer et al. 1984, Enevold et al. 2007), southeast Asian ovalocytosis (Cattani et al. 1987, Foo et al. 1992), glucose-6-phosphate dehydrogenase (Mombo et al. 2003), and blood group O polymorphisms (Facer & Brown 1979, Martin et al. 1979, Shimizu et al. 2005) are associated with protection from malaria by reducing parasitic densities.

## Innate immunity

It has been reported that neutrophil antibody-dependent respiratory burst (ADRB) activity is correlated with acquired disease resistance to malaria in endemic regions (Joos et al. 2010). In this study, individuals with high ADRB indexes were 17-fold less susceptible to malaria attacks than those without high ADRB activity, and this ADRB activity was dependent on intact merozoites and IgG opsonins but not on parasitized erythrocytes or complement (Joos et al. 2010). Interestingly, the production of reactive oxygen species (ROS) by neutrophilic ADRB in response to*P. falciparum* antigen-specific IgGs was extracellular and indicated a key role for CD32/FcγRII; however, the production of ROS in response to whole merozoites was almost completely within the cell, suggesting that the underlying mechanism was phagocytosis (Kapelski et al. 2014). The innate response to infected red blood cells (RBC) is also related to the functional activity of monocytes (MO) through their phagocytic activity, parasite killing through antibody-dependent cellular inhibition (ADCI), and supplying of peripheral tissues with macrophage and dendritic cells (DCs) (Chimma et al. 2009). Further, individuals with the CD14^hi^CCR2^+^CX3CR1^+^ MO subset and the highest mean levels of ADCI activity had lower blood parasitaemia levels, suggesting an antiparasitic activity associated with protection against malaria (Chimma et al. 2009). The induction and maintenance of B and T-cell responses requires functional DCs; these cells also have an important role in malaria immunity, and it was recently described that DCs from individuals with asymptomatic *Plasmodium* infection have higher expression of human leukocyte antigen-DR, which is required for antigen presentation (Kho et al. 2015). In a similar manner in a rodent model, DCs from nonlethal infections were fully functional and capable of secreting cytokines and stimulating T-cells compared to DCs from lethal infections, suggesting a major role for this cell in disease outcome and immunity (Wykes et al. 2007). Cells of the innate immune response are the first line of human defence against pathogens and may be important in control of the parasitaemia underlying cases of API.

## Adaptive immunity

The innate immune system also helps direct the response of adaptive immune cells (B and T-cells) in recognising and binding diverse antigens through a repertoire of cell surface receptors (Palm & Medzhitov 2009). It has been demonstrated that CD4^+^ and CD8^+^T-cells are important for malarial immunity in humans as well as in mouse models (Nussenzweig et al. 1967, Clyde et al. 1973, Schofield et al. 1987, Romero et al. 1989, Rodrigues et al. 1993, Tsuji et al. 1998, Hoffman & Doolan 2000, Stephens et al. 2005, Overstreet et al. 2008, Schmidt et al. 2008, Roestenberg et al. 2009, 2011, 2011, Stephens & Langhorne 2010, Friesen et al. 2010). In a clinical trial of the RTS,S/AS01E antimalarial vaccine, CD4^+^ T-cell production of TNF, with or without IFN-γ, was a potential immunologic correlate of protection against disease in individuals from an endemic area (Olotu et al. 2011). CD4^+^ cells from individuals with fewer previous episodes of malaria were more inflammatory and had greater TNF production, whereas responses from CD4^+^ T-cells from subjects with more frequent previous episodes of malaria were more typical of regulatory T-cells in that they produced IL-10 (Jagannathan et al. 2014). In this report, the absence of pro-inflammatory CD4^+^ T-cells producing TNF was associated with asymptomatic infection (Jagannathan et al. 2014). Thus, it suggests that IL-10 production by T-helper 1 T-cells may help prevent immunopathology by dampening the pro-inflammatory response (TNF) and preventing the development of clinical disease (Jagannathan et al. 2014).

T regulatory (Treg) cells (CD4^+^CD25^+^FOXP3^+^) appear to mediate their effects by direct cell contact or by induction of the regulatory cytokines IL-10 or transforming growth factor (TGF)-β (Thornton & Shevach 2000, Powrie et al. 2003). Treg cells are induced following *P. falciparum* and *P. vivax* infection and are associated with a burst of TGF-β production and decreased pro-inflammatory cytokine production (Walther et al. 2005, Gonçalves et al. 2010). Nevertheless, exposed asymptomatic controls (with or without parasitaemia) in a malaria-endemic region of Indonesia had a lower frequency of Treg cells (CD4^+^CD25^+^Foxp3^+^CD127^lo^) than did patients with uncomplicated and severe malaria, suggesting a role for Treg reduction in malaria protection (Minigo et al. 2009). Intriguingly, increased expression of TNFRII, a marker of Treg activation, was found in Treg cells from API subjects when compared with uninfected individuals, a feature that might be important for survival of the parasites in asymptomatic carriers; however, TNFRII expression was not measured in patients with mild or severe malaria (Wammes et al. 2013). Congolese children with asymptomatic infection have a higher prevalence of polymorphisms in regulatory genes (*STAT6* and *IL10RA*), which may influence Treg cells and malaria protection (Koukouikila-Koussounda et al. 2013).

The humoural response is also important for malaria protection because passive transfer of IgG from immune African adults to children and nonimmune adults with acute malaria rapidly reduces parasitaemia and abrogates fever (Cohen et al. 1961, Sabchareon et al. 1991). Not all exposure to malaria results in the generation of memory B-cells (MBCs) and IgG antibodies against *P. falciparum* are short-lived and fail to boost upon re-infection. Thus, immunological memory is a challenge in many vaccine trials (Dorfman et al. 2005, Bejon et al. 2006). Previous studies have described an atypical MBC population (characterised by the expression of FcRL4 and hyporesponsiveness) that is expanded in *P. falciparum-*exposed adults and children from Mali when compared with healthy United States of America controls, suggesting that this atypical population may contribute to the delayed acquisition and short-lived nature of malarial B-cell immunity (Weiss et al. 2009). Recently, it was described that atypical MBCs appear to differentiate from classical MBCs, and express a repertoire of inhibitory receptors and a deficient B-cell receptor signalling, which leads to impaired B-cell proliferation, cytokine production, and antibody secretion (Portugal et al. 2015). Other B-cells subtypes also seem to influence malaria resistance as Portugal et al. (2012) demonstrated that the percentage of activated MBCs and plasma cells was higher in the resistant Fulani ethnic group compared to those in the susceptible Dogon ethnic group, suggesting a role for B-cells in the protective immunity observed in the Fulani individuals. Individuals with asymptomatic infection tend to have higher titres of *P. falciparum* antigen-specific IgG than do individuals with other malaria outcomes. This higher response has been described as specific to several antigens, such as *P. falciparum*rifin on the surface of RBCs, recombinant protein fragments of *P. falciparum* rhoptry-associated protein-1, *P. falciparum*merozoite protein (C-terminal 10 kD), *P. falciparum* CLAG 9 (composed of 3 subunits named RhopH1, RhopH2, and RhopH3), and malaria-infected erythrocyte variant surface antigens, including *P. falciparum*erythrocyte membrane protein 1, *P. falciparum* merozoite surface protein 1 3D7 (MSP142), *P. falciparum* VarO rosetting variant, and*P. falciparum* erythrocyte binding-like and reticulocyte binding-like proteins (Alifrangis et al. 1999,Braga et al. 2002, Abdel-Latif et al. 2003, Kinyanjui et al. 2004, Villasis et al. 2012, Costa et al. 2013, Moormann et al. 2013, Sagna et al. 2013). Further, high antibody levels against glycosylphosphatidylinositols, the anchor molecules of some membrane proteins of*Plasmodium* species, is also observed more frequently in children with asymptomatic infections than in children with symptomatic infections in The Gambia (de Souza et al. 2002). Asymptomatic malaria carriers were also associated with high antibody levels against human brain antigens and *Escherichia coli* proteins as a result of polyclonal immunoglobin reactivity (Fesel et al. 2005). Furthermore, our group described that higher titre of IgG antibody against *Anopheles darlingi* mosquito saliva is also associated with immunity in asymptomatic *P. vivax* individuals from the Brazilian Amazon Region as a result of higher exposure to the malaria vector (Andrade et al. 2009). The intense production of antibodies in asymptomatic malaria carriers represents an active immune response and highlights the role of the humoural immune response in mediating disease resistance.

## Biomarkers

A biomarker is any parameter that can be used as an indicator of a particular disease state or other physiological state and can be generally classified as either biomarkers for diagnosis or for disease severity (Andrade & Barral-Netto 2011). In the context of API, biomarkers can help investigators understand disease pathology by measuring important parameters in various immune pathways and may also be useful as markers of prognosis in either clinical or silent infection after *Plasmodium* sp. exposure (Laishram et al. 2012). In recent years, our group and others have been searching for human genetic factors and plasma measures related to the immune response associated with asymptomatic infection. However, none of these factors was sufficiently powerful to be a prognostic surrogate marker of clinical protection or disease susceptibility (Andrade & Barral-Netto 2011, Mendonça et al. 2012b).

Laboratory measures are commonly used in medical practice as organ dysfunction parameters; individuals with asymptomatic *P. vivax* malaria have lower levels of aspartate aminotransferase (AST), alanine aminotransferase (ALT), indirect bilirubin, and serum creatinine as well as higher levels of Hb than do individuals with mild or severe symptomatic *P. vivax* malaria (Andrade et al. 2010b). TNF is a pro-inflammatory cytokine that has attracted special interest because of its ambiguous activity in host defence and in the pathogenesis of cerebral malaria and other severe complications (Kwiatkowski 2000). An increased TNF concentration is associated with symptoms of mild malarial pathogenesis (i.e., fever) as well as severe forms of infection, such as cerebral malaria (Kwiatkowski et al. 1990, Karunaweera et al. 1992). However, TNF-α has also been associated with the presence of potent antiparasitic activity as persistently elevated levels of this cytokine lead to rapid improvement of fever and reduction of parasitaemia (Mordmüller et al. 1997, Depinay et al. 2011). It is also noteworthy that patients with asymptomatic *P. vivax* malaria have lower levels of pro-inflammatory TNF and IFN-γ and higher levels of IL-10, a trend which is proportional to disease severity (asymptomatic, mild, and severe) and which may explain the immunological control of clinical disease. However, parasite burden control may involve a more complex host response in addition to the moderation of TNF levels (Andrade et al. 2010b, Mendonça et al. 2013). In another setting in the Brazilian Amazon, it was found that asymptomatic carriers of low *P. vivax* parasitaemias also had lower levels of TNF and IFN-γ than did symptomatic *P. falciparum* or *P. vivax*subjects (Gonçalves et al. 2012). Furthermore, certain combinations of genotypes in inflammatory-related genes (*DDX39B*, *TNF* and *IL6*) are associated with a decreased risk of mild malaria compared to asymptomatic infection by reducing plasma levels of IL-6 and TNF (Mendonça et al. 2014).

The immune and organ dysfunction response during malaria may be a result, at least in part, of the harmful effects of free haem in the human host (Gozzelino et al. 2010). During parasite-induced intravascular haemolysis, great amounts of Hb are liberated; in the presence of superoxide and other ROS, Hb releases its haem prosthetic group (Bunn & Jandl 1968, Hebbel et al. 1988, Pamplona et al. 2007, Ferreira et al. 2008). Free haem is a harmful molecule and can cause cytotoxicity, inflammation, oxidative stress, and even cell death (Ferreira et al. 2008, Gozzelino & Soares 2011). Free haem levels exhibit a linear increase according to disease severity in asymptomatic *P. vivax*-infected subjects with the lowest haem plasma concentrations (Andrade et al. 2010a). In addition, haem is also elevated with malaria severity by *P. falciparum*, especially for cerebral malaria and acute renal failure subjects (Dalko et al. 2015). In addition to enhancing pro-inflammatory mechanisms, free haem during *P. vivax* malaria also impairs prostaglandin E2 (PGE2) and TGF-β production through superoxide dismutase (SOD)-1-dependent mechanism (Andrade et al. 2010a). SOD-1 is also elevated proportionally with disease severity in malaria patients and is useful for distinguishing mild and asymptomatic *P. vivax* cases by ROC curve analysis (Andrade et al. 2010c). In addition, asymptomatic carriers have higher concentrations of regulatory cytokines such as TGF-β and PGE2 compared with mild and severe *P. vivax* patients, and TGF-β and PGE2 are negatively correlated with SOD-1, which may be an additional defence mechanism against disease manifestation (Andrade et al. 2010a). In*P. falciparum* malaria, bicyclo-PGE2 is also elevated in asymptomatic patients compared with patients who have symptomatic disease (Perkins et al. 2005).

Over time, the human host has evolved protective mechanisms against the deleterious effects of free haem in the circulation. When Hb is released from ruptured RBC upon*Plasmodium* sp. infection, it is scavenged by haptoglobin (Hp) and prevents the release of haem. The complex Hp-Hb is recognised by CD163 on the macrophage and hepatocyte surfaces in the spleen and liver, respectively (Philippidis et al. 2004, Quaye 2008). Free haem can also be scavenged by haemopexin, albumin, α1-microglobulin, and high and low-density lipoproteins (Bunn & Jandl 1966, Miller & Shaklai 1999, Paoli et al. 1999, Allhorn et al. 2002, Fasano et al. 2007, Tolosano et al. 2010). Different Hp phenotypes are known to have different binding affinities for cell-free Hb (Hp1.1>Hp1.2>Hp2.2) and CD163 (Hp2.2>Hp1.2>Hp1.1) (Kristiansen et al. 2001). Our group has reported that individuals with the *Hp2* allele are more likely to have symptomatic*P. vivax* malaria, and this group also has higher levels of Hp when compared with those of patients with asymptomatic infection. This probably represents a compensatory mechanism against the low binding affinity of*Hp2* to free Hb (Mendonça et al. 2012b). The Hp2.2 phenotype has also been associated with a higher susceptibility to *P. falciparum* infection in the Dogon ethnic group living in Mali (Perdijk et al. 2013). Furthermore, soluble CD163 (sCD163) (marker of receptor activation) is also lower in asymptomatic patients when compared with that in symptomatic subjects, and a cut-off value of sCD163 may be used to distinguish between symptomatic and disease-free individuals (Mendonça et al. 2012b). In Mali, sCD163 was increased in *P. falciparum* infected individuals compared to uninfected subjects (Perdijk et al. 2013). Inside the cell, haem is degraded by HO-1 to produce carbon monoxide (CO), labile iron, and biliverdin. In murine models, HO-1 affords protection against cerebral malaria by reducing neuroinflammation (including CD8^+^ T-cell brain sequestration), and exposure to CO may reduce severe complications (Pamplona et al. 2007). HO-1 also seems to be one of the mechanisms by which sickle cell disease confers protection against experimental malaria (Ferreira et al. 2011). HO-1 plasma levels are higher in symptomatic cases (as compared to asymptomatic individuals) as a regulatory defence, and a microsatellite polymorphism (GT)n in*HMOX1* regulates the expression of this enzyme (Mendonça et al. 2012b). In addition, high HO-1 levels and this microsatellite polymorphism were associated with severe malaria, including death, in another study (Walther et al. 2012). However, other studies also have demonstrated conflicting results and no association between this *HMOX1* microsatellite polymorphism and malaria severity (Kuesap et al. 2010 ,Hansson et al. 2015).

Iron is produced by haem catabolism and also obtained by dietary uptake; this metal is necessary for complete *Plasmodium* development (Gozzelino et al. 2010). However, intracellular labile iron is dangerous because it converts to a free radical unless it is scavenged by ferritin, which acts as a vital antioxidant molecule in several experimental models (Balla et al. 1992, Cozzi et al. 2000, Berberat et al. 2003). Ferritin serum levels are decreased and associated with anaemia in a population from the Brazilian Amazon exposed to*P. vivax* malaria; symptomatic individuals from this group infected with *P. vivax* have lower levels of ferritin, which are directly proportional to the hepatic damage score (Cardoso et al. 1994, Gozzelino et al. 2012). It has been reported that ferritin promotes disease resistance to malaria by preventing labile intracellular iron from sustaining pro-apoptotic c-Jun N-terminal kinase activation, and this tolerance requires the expression of HO-1 (Gozzelino et al. 2012). Interestingly, malarial tolerance mediated by ferritin production is independent of the parasitaemia rate and represents a host defence strategy to limit the fitness costs of infection irrespective of pathogen burden (Medzhitov et al. 2012).

## New approaches to understanding asymptomatic infection

In recent years, large amounts of data have become available as a result of the progress in technological methods, such as multiplex measurements, genome-wide genotyping, microarrays, RNAseq, and multicolour flow cytometry (Tran et al. 2012). Genome-wide studies allowed the discovery of important *loci* related to malaria resistance and low parasitaemia. Linkage of asymptomatic parasitaemia to 5q31-q33 has been reported in humans (Rihet et al. 1998, Timmann et al. 2007) and, recently, chromosomes 6p21.3 and 17p12 were correlated with resistance in individuals from Burkina Faso (Brisebarre et al. 2014). Equally important, the field of engineered humoural immunity (with the production of human monoclonal antibodies) has allowed a better understanding of the malaria immune response by facilitating several laboratory methods (i.e., multiparameter flow cytometry).

To understand this large volume of information, new approaches for data analysis have become more widespread and multivariate (clusters, principal component analysis, etc.), artificial neural, Bayesian, and network analysis methods are some tools that can be used to characterise a molecular signature of resistance or susceptibility to malaria (Jayavanth & Singh 2003, Kiang et al. 2006, d, da Cunha et al. 2010, Bachtiar et al. 2013). Many studies have attempted to identify molecular signatures associated with severe *P. falciparum* malaria, but few have focused on the mechanisms behind asymptomatic *Plasmodium* infection (Timmann et al. 2007, 20, 2012, Jallow et al. 2009, Milet et al. 2010). Using a network approach, our group recently described the interactions among cytokines, chemokines and other inflammatory proteins associated with different *P. vivax* malaria outcomes (Mendonça et al. 2013). Network analysis allows a better understanding of the inflammatory profile from different malaria groups by allowing easy visualisation of interactions between several markers and identification of patterns of association that may indicate susceptibility or disease tolerance signatures. Using network analysis, it has been demonstrated that patients with asymptomatic *P. vivax*malaria have an overall reduction in pro-inflammatory cytokines (TNF, IFN-γ, IL-6) and markers of tissue damage (ALT, AST, creatinine, bilirubin, and others) and augmented levels of regulatory cytokines (TGF-β and IL-10) when compared with those of the symptomatic groups (mild and severe malaria) (Mendonça et al. 2013). Furthermore, IL-4 had the highest number of interactions between all the markers in the asymptomatic group, suggesting a possible role for this cytokine in mediating *P. vivax* malaria tolerance (Mendonça et al. 2013). Others studies have also used the same network analysis for placental malaria and malarial anaemia, but none analysed asymptomatic infection (Ong’echa et al. 2011, Sikora et al. 2011). In this context, cohort studies with a large sample size and an extensive bioinformatics approach are highly necessary to better understand the interactions among the immune response pathways associated with asymptomatic infection tolerance.

## Concluding remarks

**Fig. 2:**
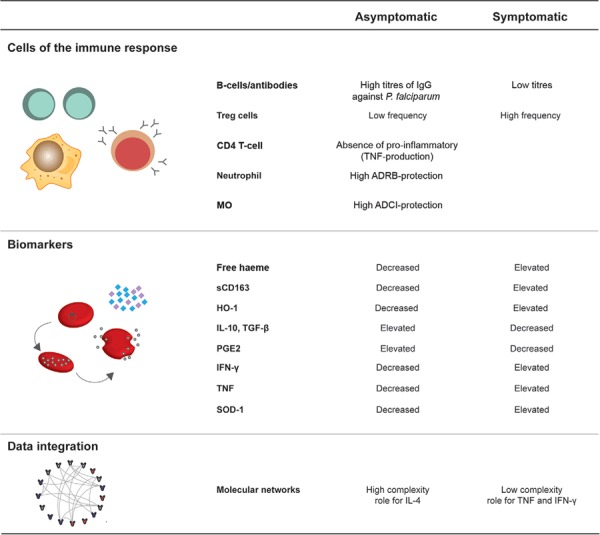
: the immune response underlying asymptomatic infection. Aspects of the immune response of asymptomatic malaria carriers were compared to symptomatic patients. This response was didactically divided into immune cells including T regulatory (Treg) cells, CD4+ T-cells, B-cells, neutrophils, and monocytes (MOs) and biomarkers related to inflammation [interleukin (IL)-10, transforming growth factor (TGF)-β, prostaglandin E2 (PGE2), interferon (IFN)-γ, and tumour necrosis factor (TNF) and the haeme pathway [haeme, soluble CD163 (sCD163), haeme oxygenase-1 (HO-1), and superoxide dismutase (SOD)-1]. Additionally, molecular networks in the context of asymptomatic infection illustrate the use of methods of data integration in immunology. ADCI: antibody-dependent cellular inhibition; ADRB: antibody-dependent respiratory burst.

It is noteworthy that API is related to clinical disease tolerance (i.e., absence of symptomatology) but is not associated with immunity and inflammatory tolerance. Asymptomatic *P. vivax* infection is an active and acquired state, and it can control parasitaemia and limit organ dysfunction by an as yet poorly understood immune mechanism. Asymptomatic individuals carrying the parasite are natural reservoirs representing a challenge for malaria eradication, primarily in low and moderate-endemic countries. The use of mass drug administration or mass screening and treatment schemes is controversial (Tada et al. 2012). Overall, biomarkers related to the haem pathway and iron metabolism have emerged in recent years as potential clues to unravel the immune response of API. Despite this progress, there is no reliable marker of prognosis in API. Immune cells, especially Tregs and B-cells, seem to play an important role in protection from disease manifestation. Furthermore, it has been observed that the immune response in individuals with asymptomatic infection is usually associated with a lower pro-inflammatory and a higher regulatory production of biomarkers and host genetic alterations that may contribute to malaria tolerance. Nevertheless, the acquisition of large-scale biological data along with the use of robust bioinformatics tools, including a network approach, will help investigators to understand the immune response behind asymptomatic infection. The major topics described here are summarised in Fig. 2. Longitudinal studies of sequential episodes of malaria in the same individual are necessary to better understand the immune response of individuals with API who are able to clear their parasitaemia compared with those who are more likely to have a symptomatic disease or remain symptomless despite the presence of*Plasmodium* sp. With this understanding, better medical management of API carriers, the development of malarial vaccines, and strategies for malaria eradication will be facilitated.
